# Plasmonic nanostructures fabricated using nanosphere-lithography, soft-lithography and plasma etching

**DOI:** 10.3762/bjnano.2.49

**Published:** 2011-08-16

**Authors:** Manuel R Gonçalves, Taron Makaryan, Fabian Enderle, Stefan Wiedemann, Alfred Plettl, Othmar Marti, Paul Ziemann

**Affiliations:** 1 Ulm University, Institute of Experimental Physics, Albert-Einstein-Allee 11, 89069 Ulm, Germany; 2 Ulm University, Institute of Solid State Physics, Albert-Einstein-Allee 11, 89069 Ulm, Germany

**Keywords:** nanosphere-lithography, near-field enhancement, plasma etching, soft-lithography, surface plasmons

## Abstract

We present two routes for the fabrication of plasmonic structures based on nanosphere lithography templates. One route makes use of soft-lithography to obtain arrays of epoxy resin hemispheres, which, in a second step, can be coated by metal films. The second uses the hexagonal array of triangular structures, obtained by evaporation of a metal film on top of colloidal crystals, as a mask for reactive ion etching (RIE) of the substrate. In this way, the triangular patterns of the mask are transferred to the substrate through etched triangular pillars. Making an epoxy resin cast of the pillars, coated with metal films, allows us to invert the structure and obtain arrays of triangular holes within the metal. Both fabrication methods illustrate the preparation of large arrays of nanocavities within metal films at low cost.

Gold films of different thicknesses were evaporated on top of hemispherical structures of epoxy resin with different radii, and the reflectance and transmittance were measured for optical wavelengths. Experimental results show that the reflectivity of coated hemispheres is lower than that of coated polystyrene spheres of the same size, for certain wavelength bands. The spectral position of these bands correlates with the size of the hemispheres. In contrast, etched structures on quartz coated with gold films exhibit low reflectance and transmittance values for all wavelengths measured. Low transmittance and reflectance indicate high absorbance, which can be utilized in experiments requiring light confinement.

## Introduction

Classical electromagnetic theories describing optical transmission through small apertures [1–2] do not take into account the role of surface plasmons on metal films. In contrast to the predictions of these theories, enhanced optical transmission (EOT) was found for arrays of holes in metal films [3]. The transmission enhancements were attributed to the surface plasmons excited in the array [4]. This discovery triggered extensive research on nanostructures that support surface plasmons, namely, nanocavities on metal films, arrays of interacting metal particles and gratings. The coupling between light and localized surface plasmons on metal nanostructures that have been favorably tailored leads to a variety of effects, such as optical resonances [5–10], near-field enhancements [11–14], enhanced scattering [15], enhanced transmission [3–4,16–24], and plasmonic whispering gallery modes [25–27]. Some of these effects have been explored in applications such as surface enhanced Raman spectroscopy (SERS) [28–31] and, more recently, in studies of fluorescence lifetime [32–33] and the enhancement of the Purcell rate [34] (achieved mainly by confinement of light in small mode volumes rather than by very large *Q*-values of the resonances).

The strong sensitivity of these effects to the shape and size of the structures means that we require good reproducibility in the fabrication technique and good knowledge of the optical properties. However, some applications demand structures of extended size. Thus, the optimization of fabrication methods is intimately linked with the optical function of the structures.

Current techniques for the fabrication of plasmonic cavities include electrochemical growth combined with nanosphere lithography [25,35], electron-beam lithography [36], etching techniques [37–40] and focused ion beam milling [41–43]. The techniques based on electron beam lithography and focused ion beam milling allow us to obtain structures of arbitrary shape and two-dimensional profile, but they are size limited and time consuming.

Applications outside of sensing are also envisaged. Plasmonic resonators can not only confine light but can also enhance scattering at their resonances. This effect has been exploited in solar cells, for example, enhanced scattering by arrays of silver nanoparticles permits a thickness reduction of Si solar cells without compromising the intrinsic energy conversion efficiency [44–46]. Applications of this kind require large area nanostructured surfaces. Thus only methods allowing large scale lithography/patterning are appropriate for this purpose. E-beam lithography and FIB based nanofabrication would be prohibitively expensive.

The most common fabrication technique using arrays of polystyrene (PS) or silica beads is based on the evaporation of metal films on top of the spheres. This technique is called nanosphere lithography and the patterns obtained on the substrate are often referred to as Fischer’s projection patterns [47]. Nanosphere lithography can serve as a large scale fabrication method and the lattice constant of the resulting structures can be changed by adequate choice of the diameter of the beads [35,48]. The standard shape of the projected pattern is triangular, but etching techniques have been used to obtain other shapes [49].

EOT was investigated from polystyrene or silica spheres coated by metal films [50–53]. The interstices of the coated beads form an array of triangular holes on a corrugated surface. The details of the pattern projected on the substrate have no significant effect on the optical transmission according to calculations [51]. However, plasmonic structures fabricated by nanosphere lithography can also be used for other purposes.

Soft lithography [54] is an alternative technique for nano- and micro-fabrication involving the inverse replication of a mold with the aid of elastomeric polymers. It can be reliably scaled down to sizes of ~100 nm. Nanoimprint lithography [55–56] is another alternative technique in which a pattern is formed on top of a substrate by pressing a mold against a thin resist film, followed by reactive ion etching (RIE) of the patterned substrate. This allows patterning of reproducible structures up to a few tens of nanometers. However, instead of casting the resist for preparation of the mask for RIE, high ordered arrays of PS spheres can be used directly.

With this in mind, we propose two fabrication routes to obtain periodic structures comprising arrays of nanocavities in metal films. Both techniques are suitable for large scale fabrication. The optical properties of these structures can be exploited in applications requiring strong confinement of light.

## Results and Discussion

**Figure 1:**
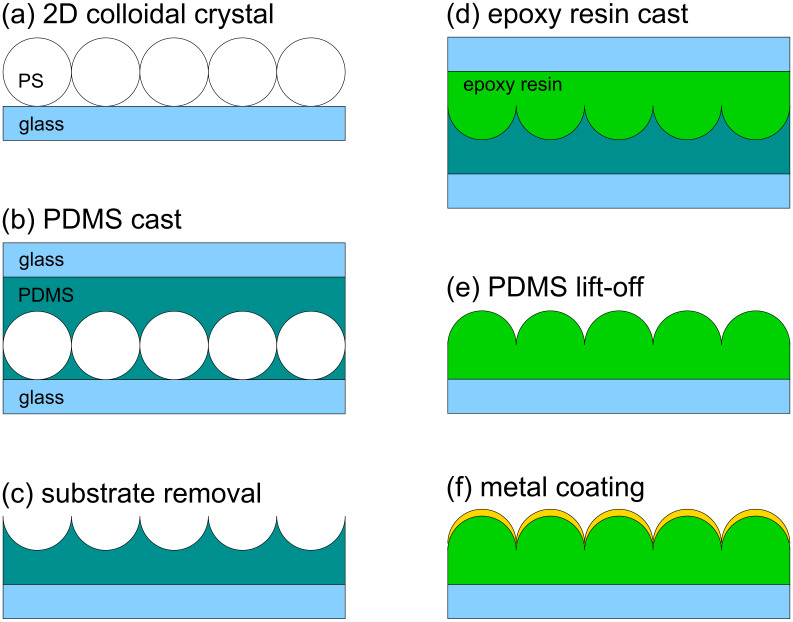
Fabrication of arrays of metal film coated hemispheres. Main steps: (a) Preparation of 2D colloidal crystal; (b) cast of polydimethylsiloxane (PDMS); (c) detachment of substrate; (d) cast with epoxy resin; (e) PDMS detachment and (f) metal coating by physical vapor deposition.

The fabrication techniques comprise several steps, including preparation of colloidal crystal templates, metal evaporation and one or more casting steps. The most important steps for the fabrication of metal coated hemispheres are presented in Figure 1. The main steps in the etching of quartz substrates, patterned with hexagonal arrays of Cr triangular particles to obtain arrays of triangular mesas or triangular holes, are depicted in Figure 4.

**Figure 2:**
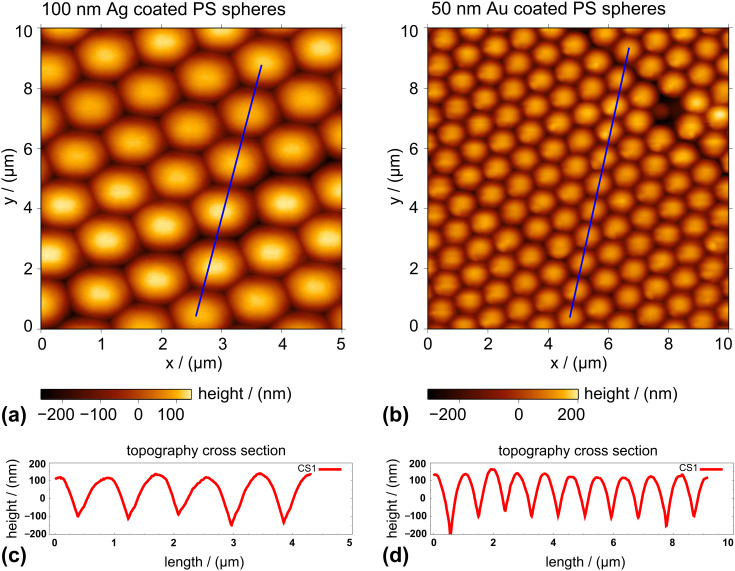
AFM topography images of coated beads (a) and coated hemispheres (b), both fabricated using PS spheres of 1 μm diameter. The plots of (c) and (d) are cross sections of lines marked in the topography images.

The topography of the structures at different stages of fabrication was characterized by atomic force microscopy (AFM) (Figure 2, Figure 5 and Figure 7) and by SEM (Figure 6). The optical measurements performed on coated hemispheres, polystyrene (PS) spheres and on coated arrays of pillars are discussed in the next two subsections.

### Coated hemispheres

Figure 1 shows schematically the fabrication of hemispheres coated by a metal film. After the preparation of a PS 2D colloidal crystal and two casting steps, the resulting structures were metal coated by physical vapor deposition. In Figure 2, AFM images of coated beads and coated hemispheres are shown. For further details on the fabrication, see the Experimental section.

Reflectance and transmittance measurements on coated spheres and hemispheres of the same size permit a comparison of the resonances and their spectral positions. While in the case of coated spheres there are interstices serving as transmission holes, in the case of hemispheres there are no regularly spaced holes. Thus, on thin gold films only low transmission can be expected. However, the coated hemispheres can be seen as a two-dimensional grating with deep “valleys”.

**Figure 3:**
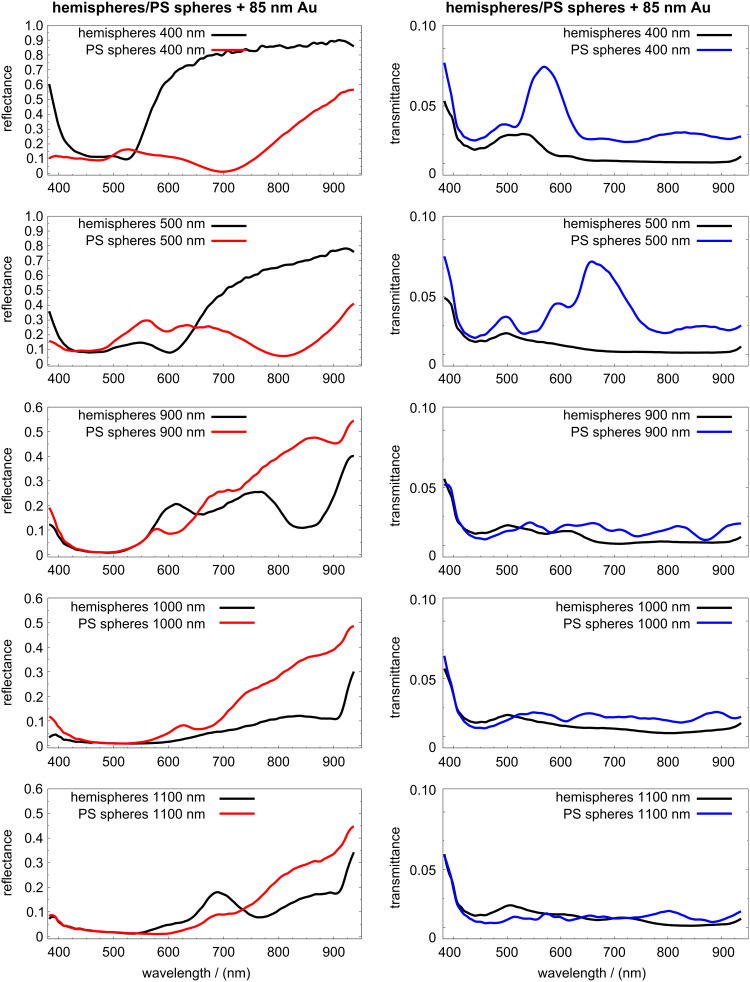
Reflectance (left) and transmittance (right) obtained at vertical illumination with an objective of *NA* = 0.25. The diameter of the PS spheres or hemispheres is indicated in the legend of each spectrum. The thickness of the gold film is approximately 85 nm.

Significant differences were observed for the reflectance measured on comparing gold coated PS spheres to hemispheres, within the size range of 400 nm to 1100 nm (Figure 3). The array of spheres exhibited low reflectance bands corresponding to wavelengths of around 700 nm (for 400 nm diameter) and 800 nm (for 500 nm). In contrast, there were no such low reflectance bands for coated hemispheres. The low reflectance band between 400 nm and 500 nm wavelengths (on the 400 nm and 500 nm diameter coated spheres) broadens for spheres of large size. The first peak appearing at around 570 nm (for 900 nm spheres) was shifted to higher wavelengths with increasing sphere diameter.

Hemispheres of 400 nm diameter coated with 85 nm Au exhibited a lower reflectance than that of the coated spheres of around 525 nm diameter. This was more evident for 500 nm diameter hemispheres, where the band of low reflectance extended from 500 nm to 650 nm. Simultaneously, the transmittance of coated hemispheres, mainly those of 400 nm and 500 nm diameter, was clearly below that of the coated spheres. In contrast, the transmittance spectra of the coated spheres showed resonances at λ ≈ 570 nm (for 400 nm spheres) and at λ ≈ 670 nm (for 500 nm spheres).

Larger hemispheres (with diameters of 900 nm and 1100 nm) presented more complex optical spectra, with bands of both higher and lower reflectance compared to those of the spheres. The zeroth-order reflectance of 1100 nm hemispheres was, in general, lower than that of the spheres with the same diameter. The transmittance for the last three diameters was similar for both structures. However, the coated hemispheres generally transmitted less light than the coated spheres. Some of our transmittance results can be compared with the results from experiments performed with silver coated spheres of different sizes [51]. The transmission spectrum of PS spheres of 390 nm diameter, coated with 75 nm Ag, presented a similar spectral resonance to that of 400 nm spheres coated with 85 nm Au, but the peak was shifted to a lower wavelength. In general, samples of Ag coated spheres exhibited higher transmittance than Au coated ones. The position of the highest transmittance found in PS spheres of 400 nm and 500 nm was shifted to higher wavelengths. The highest transmittance for spheres of larger diameter (900 nm and above) is expected to be found at wavelengths above 1000 nm.

The low reflectance bands of Au coated hemispheres and their simultaneous low transmittance indicate a high absorbance, at least for 400 nm and 500 nm hemispheres. Under illumination at normal incidence no diffraction occurred for wavelengths smaller than the diameter of the hemispheres. An increase in diffraction intensity in some or all diffraction orders, at the expense of the zeroth-order reflectance, cannot be excluded for the three largest diameters. However, the shift to higher wavelengths of the low reflectance bands for the larger structures cannot be explained exclusively by diffraction effects. In particular, for the 900 nm hemispheres there is a band, around 600 nm, where the reflectance was larger than that for the sample with coated spheres. For the 1000 nm hemispheres the reflectance remained always below the corresponding value for the coated spheres.

According to some reports, both total absorption [57] and omnidirectional absorption [58] can occur on nanostructured metal surfaces at certain wavelengths. Indeed, we found spectral bands of very low reflectance and low transmittance for 400 nm and 500 nm diameter PS spheres coated with gold, at around λ = 700 nm and λ = 800 nm, respectively. Therefore in these bands the absorbance must be quite large.

### Triangular mesas and holes

**Figure 4:**
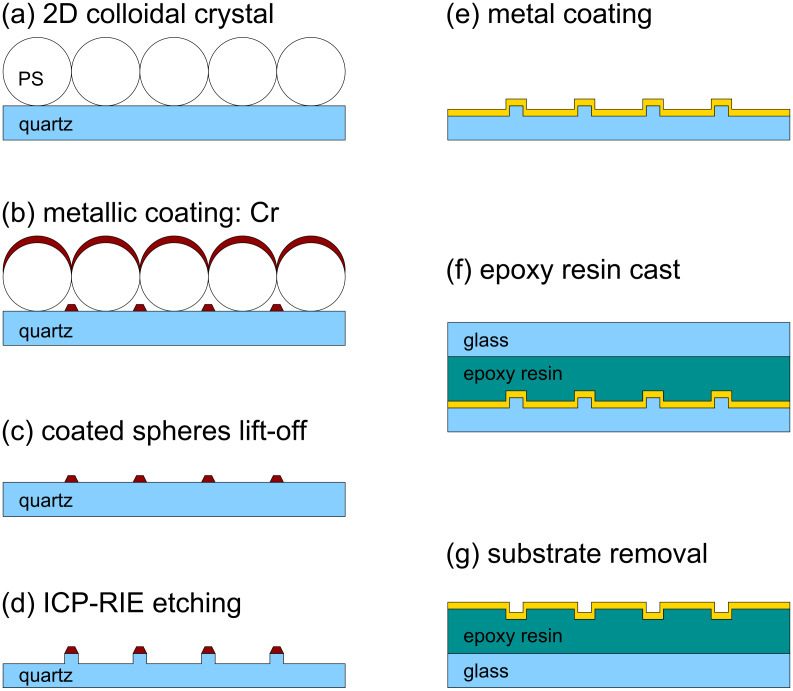
Fabrication steps for arrays of triangular mesas etched in quartz and arrays of holes in metal films, prepared by casting with epoxy resin: (a) Preparation of 2D colloidal crystal; (b) Cr coating; (c) PS spheres lift-off; (d) RIE; (e) metal coating (PVD) (f) epoxy resin cast; (g) substrate removal.

**Figure 5:**
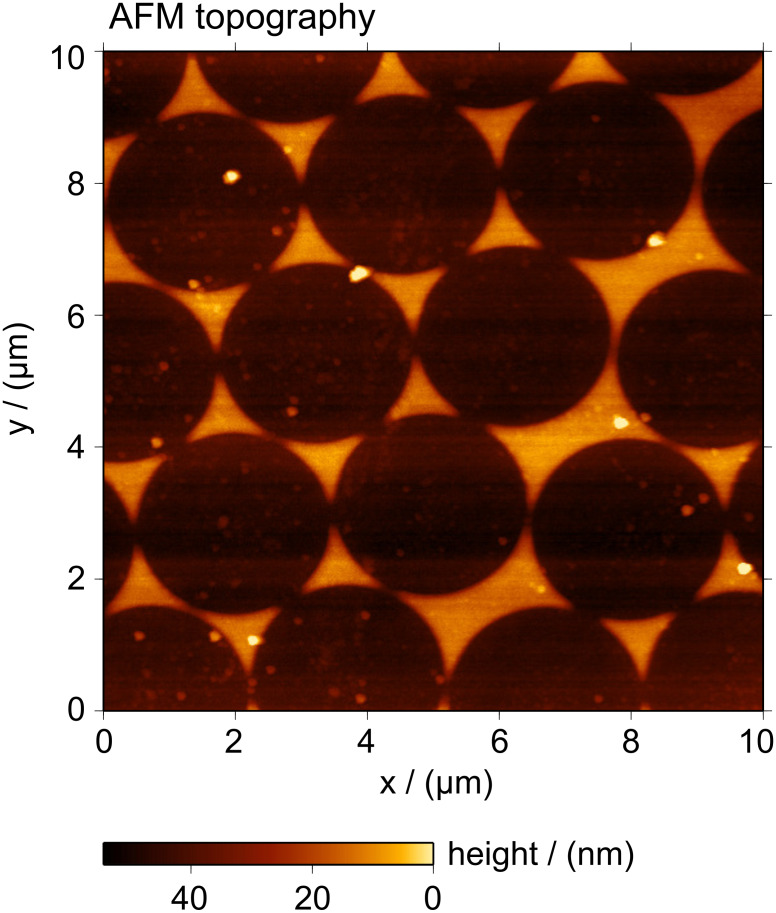
Topography image of a projection pattern of Cr on quartz. The height of the pattern is 30 nm. Small particles scattered on the surface are chemical species that were not completely removed during the spheres lift-off.

The main steps for the fabrication of arrays of quartz triangular mesas and arrays of triangular holes, obtained by epoxy resin cast, are presented in Figure 4. In order to produce the masks for reactive ion etching, a film of chromium was evaporated on top of the PS beads. The mask obtained was in the form of a hexagonal array of triangular structures. Measurements of the topography by atomic force microscopy (WITec Alpha 300 AFM in AC mode) typically gave larger thickness values than those given by the quartz crystal balance; Figure 5 shows an example of such a measurement. Some protrusions and isolated clusters seen in the AFM image are due to residual polymer material or other chemical species that were not completely removed by the cleaning process.

**Figure 6:**
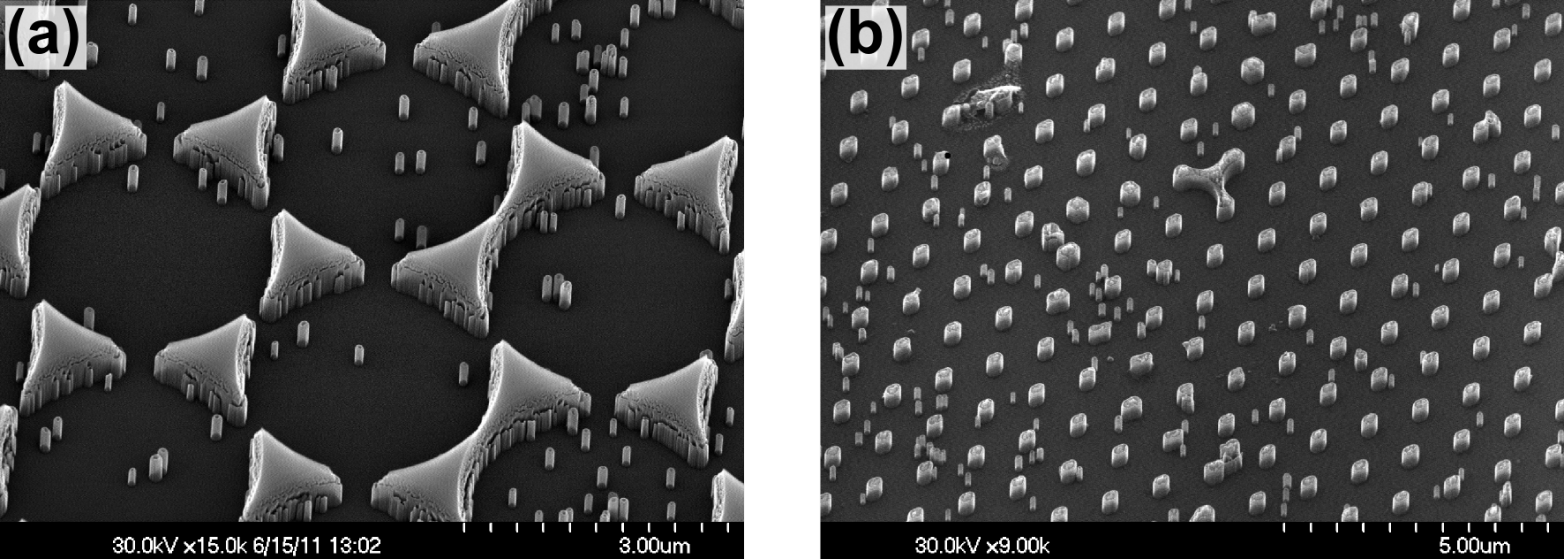
SEM micrographs of arrays of triangular mesas etched into the quartz substrate. Spheres of 3 μm diameter (a) and 1 μm (b) were used for the production of the Cr mask. The pattern of the etched mesas in the right image was obtained from a Cr deposition over a double layer of spheres. The depth of the quartz structures was (a) 500 nm and (b) 300 nm. The thickness of the Cr film of the mask was (a) 30 nm and (b) 18 nm.

Scanning electron micrographs of samples of etched quartz, fabricated from Cr masks of 3 μm and 1 μm size, are presented in Figure 6. The left image shows triangular mesas replicating the shape of the Cr mask, and also some isolated pillars within the etched substrate area. The reason why the pillars were formed has not yet been determined, but it may be due to residual contaminants on the quartz surface. Deep etched structures (500 nm) exhibited rough side walls near the triangular Cr pattern (Figure 6a). Increasing the thickness of the Cr film produced patterns that were not terminated by vertical sidewalls but instead with tails. Thus, the roughness can be explained by progressive erosion of the Cr tails during the RIE. On thinner Cr films (18 nm) the side walls of the etched pattern were smoother and more vertical (Figure 6b).

In order to avoid problems associated with the formation of the small isolated pillars within the otherwise flat area, an additional ion bombardment step had to be implemented. For this purpose, Ar^+^ sputter cleaning (3.5 μA/cm^2^, 3 kV, at a grazing angle of 10°) for typically 20 min proved to be suitable, as demonstrated in the right electron microscopy image of Figure 6. Compared to etched samples without this Ar^+^ cleaning step, the amount of random pillars and roughness is strongly reduced.

**Figure 7:**
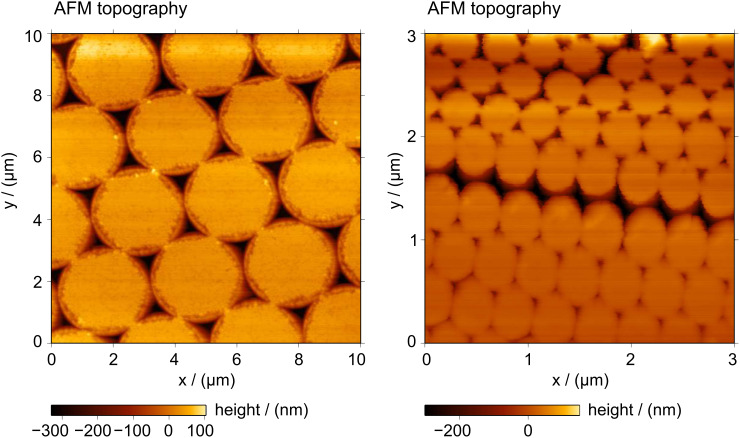
Arrays of triangular holes in 180 nm thick gold film. Monolayer colloidal crystals of 3 μm (left) and 400 nm (right) PS spheres were used as templates for the fabrication. The progressive degradation of quality in the topography of the right image is due to the reduction of the tip radius of the cantilever during scanning.

Subsequently, the RIE the Cr masks were removed by wet etching. The surface of etched quartz is hydrophilic. This is not a limitation for the metal coating but does, however, impede the detachment of the casting polymers. Indeed, cured epoxy resin cast directly onto etched quartz could not be removed. One might expect that aliphatic silane molecules would bond covalently to silicon oxide, forming a hydrophobic surface. However, no significant change was observed after dipping the sample in silane solution, and epoxy resin cast on that sample could still not be detached. For the preparation of an anti-adhesive coating the same plasma etching system was used. By plasma polymerization of the process gas CHF_3_, a fluoro-carbon film was deposited on the previously prepared quartz substrate. This technique delivers layers of excellent conformity, and of very low surface energy, to the subjacent structure [59–60]. Furthermore, this coating technique works on most substrates, e.g., silicon, glass, metals, or any on passivation layer on the pillars. In all cases, the thickness of the anti-adhesive layers is in range of 5 nm to 10 nm. These additional layers allow detachment of the cured epoxy resin cast on top of etched quartz, or on top of metal films that then remain bound with the cast material. Figure 7 presents two AFM topography images of samples fabricated from 3 μm and 400 nm PS beads, respectively, with evaporation of 180 nm of Au and a cast of epoxy resin. The resulting structures have triangular holes within the gold films that were detached together with the epoxy resin. Even for the sample using 400 nm beads, where the average side of the triangular particles is of the order of 130 nm, deep triangular holes are obtained. The roughness at the edge of the triangles, caused by the pillar shaped defects previously mentioned, is still reflected in the topography of the gold film (Figure 7 left). Our preliminary results, in experiments employing colloidal crystals of PS beads, of 3 μm, 1 μm and 400 nm diameter, as templates for the etching masks, demonstrate that it is possible to tailor well-defined quartz mesas and deep triangular holes. Large scale fabrication is guaranteed by the size of the template. In Figure 7, AFM topography images of two of the samples after the detachment step are shown revealing sharp corners and edges of the holes within the gold film.

The roughness at the edges of the particles after the etching process, due to residual contaminants on the surface of the quartz, remains a problem. Preliminary sputtering with Ar^+^ ions improved the quality of the samples, as demonstrated by SEM studies. The samples studied with AFM were not sputtered with Ar^+^ ions. The high aspect ratio of some small defects (20 nm width and 500 nm height), found between the triangular mesas, indicates that it is possible to fabricate triangular cross-sectioned structures with a much higher aspect ratio. The gold evaporation was performed at a fixed angle, but full coating of the etched mesas is possible if the sample is rotated during the metal evaporation. By this way, shadows and hence pinholes in the metal film are less prone to occur.

**Figure 8:**
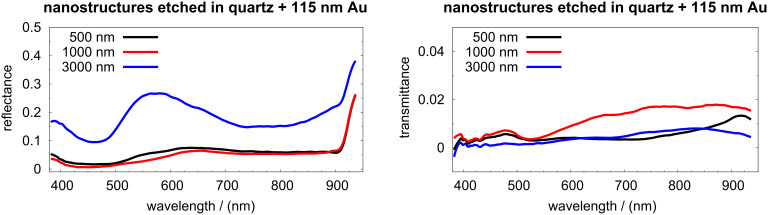
Reflectance (left) and transmittance (right) of triangular nanostructures etched in quartz and coated with 115 nm of gold. The diameter of the PS spheres used for the fabrication of the Cr masks is indicated in each spectrum.

Reflectance and transmittance spectra, of 115 nm Au films evaporated on top of structures etched on quartz, are presented in Figure 8. These films were not evaporated vertically but instead using a rotation stage with a rotation axis making an angle of 15° to the vertical. The samples were rotated during the evaporation to avoid shadows at the etched structures. However, the rotation leads to a different thickness of the film on the top of the mesas compared to on their lateral walls.

Compared to hemispheres, fabricated from spheres of the same size, the gold films evaporated on the etched quartz exhibited low reflectance and very low transmittance. The reflectance decreased with increasing pattern diameter. For 500 nm patterns no diffraction is expected for λ > 500 nm. Therefore, most of the light was absorbed. Investigations of gratings and small cavities of various shapes have demonstrated strong light confinement [10,61–65]. Thus, etched structures with sharp edges, coated by gold films are suitable to confine light. The confinement efficiency and the plasmonic mode dispersion, leading to the highest near-field enhancements, need to be investigated in more depth.

## Conclusion

Two novel methods of fabrication of plasmonic structures were introduced, based on nanosphere lithography, combining soft lithography and reactive ion etching techniques. The first permits the preparation of large arrays of hemispheres, using PS beads as templates. The second allows either the fabrication of high aspect ratio triangular mesas or, alternatively, arrays of deep holes obtained by making a polymer cast of the protrusions. The fabrication of arrays of hemispheres is relatively simple and lends itself to large scale fabrication (up to several cm^2^). PS spheres of micrometer size down to a few hundreds of nanometers can be used. However, mainly due to the low Young’s modulus of PDMS used in the fabrication of the stamp, deformed hemispheres may occur for very small PS beads. The zeroth-order reflectance and transmittance of gold films evaporated onto the hemispheres was measured. The low reflectance bands found on coated hemispheres, when compared to coated PS spheres of the same size, indicate an enhanced absorbance, which may be due to light confinement effects. Films of other materials and heterogeneous metal–insulator–metal films, of great importance in plasmonics, may be prepared in the future as well. For a better understanding, however, simulations are necessary to elucidate how light confinement occurs at the “valleys” and for which specific wavelengths. Furthermore, additional experimental methods, such as scattering-SNOM and fluorescent lifetime imaging, could provide information about local field enhancements.

The second fabrication route, involving RIE of quartz, constitutes an alternative nanofabrication method for plasmonic structures based on arrays of quartz mesas and arrays of holes in metal films. In this case the experimental aim is the preparation of large nanostructures for light confinement and, eventually, enhanced optical transmission based applications. The first transmittance and reflectance measurements indicate a strong absorbance at optical wavelengths. Here, calculations of near-fields of the prepared structures are necessary to quantify the efficiency of light confinement for various geometries. Furthermore, angular resolved reflectance spectroscopy is required as a function of the angle of incidence to confirm directional effects.

## Experimental

### Two-dimensional colloidal crystals

Colloidal suspensions of polystyrene (PS) beads of different sizes, in water, were purchased from Thermo Scientific. Sizes of 400 nm, 500 nm, 900 nm, 1000 nm, 1100 nm and 3 μm diameter were used. The suspensions conform the NIST Standards and have sharp size distributions of 1.0% to 2.5%. According to the supplier some proprietary surfactants may be added to the suspension. Volumes of 1 mL were centrifuged until full sedimentation of the beads was achieved. After removal of the liquid, the same volume of MilliQ water was added and the sediment beads resuspended. The process was repeated three times in order to remove surfactants of the suspension, which prevent the aggregation of the beads, but limit the quality and size of the two-dimensional colloidal crystals. Glass cover slides of 20 × 20 mm^2^ and quartz glass of the same size, but of 1 mm thickness (used for the fabrication of samples etched by RIE) were used as substrates. Each substrate was cleaned by sonication in methyl ethyl ketone (MEK) and isopropanol, and dried by nitrogen jet. The surfaces of the samples were submitted to an air plasma at primary vacuum for 10 s, in order to improve the wetting properties of the surface. Suspension volumes of 15 to 55 μL, depending on the size of the beads, were put on top of substrates and the samples were put inside an acrylic glass container of 1.25 cm^3^ volume. The cold sides of two Peltier elements were attached to the upper and lower sides of the sample container. By applying adequate currents the rate of evaporation was reduced and the crystallization of the beads occurred, mostly forming monolayers with areal extents up to 1 cm^2^. The number of vacancies and dislocations of the colloidal crystal increased as the size of the beads decreased. For beads of 3 μm size, single crystals of several mm^2^ were usually obtained. The evaporation of the water and full crystallization takes up to 6 h.

### Epoxy resin hemispheres

Polydimethylsiloxane (PDMS) from Dow Chemical was prepared using the elastomer and curing agent in a ratio of 10:1. The two components were mixed and air bubbles were removed by submitting the liquid to primary vacuum for 15 min. The cast of polymer beads was achieved by filling a cylindrical ring on top of the two-dimensional colloidal crystal. This limited the flow of the PDMS to the wall of the ring. The epoxy resin was cured in an oven at 35 °C for 24 h. The substrate with beads was then detached from the PDMS stamp. PS beads remaining in the PDMS were dissolved by sonication in MEK. The second cast process with epoxy resin was performed on top of the PDMS stamp (Figure 1d). Commercial epoxy resin of high transparency was used. Air bubbles were removed in vacuum, as for PDMS. A small droplet of the liquid was put on top of the PDMS and covered by a glass cover slide. The curing took 12 h at 20 °C. Afterwards the cover slide was detached from the PDMS stamp. The topography of the epoxy resin surface was a replica of the original colloidal array. Between the hemispheres there were “valleys” of approximately one beads radius in depth (Figure 1e).

### Nanostructures etched on quartz

The masks for the patterns to be etched on quartz by RIE were fabricated by nanosphere lithography and evaporation of Cr films of 5 to 15 nm thickness. The Cr coated spheres were removed by three sonications in MEK, and rinsing in MilliQ water and isopropanol. This cleaning is crucial, as chemical species adsorbed on the surface influence the anisotropic etching of quartz (Figure 5). The anisotropic RIE (using ICP-RIE Oxford Plasmanlab 80 Plus) process was applied to transfer the Cr pattern onto the quartz substrate [39–40]. The etching process uses a side wall passivation similar to the Bosch process [38], but works with a mixture of CHF_3_ and CF_4_ in a ratio of 10:1, at a working pressure of 10 mTorr. The DC bias of the etcher was set to −96 V during the process, resulting in an etching rate of approximately 4 nm/min. The same plasma etching system was used for coating of etched quartz with the anti-ahesive film. The Cr masks were removed with a commercial etching solution (Chrome Etch 1 from SOTRAMCHEM Technic, France).

### Metal coating

Gold films were deposited by physical vapor deposition (PVD), from tungsten boats, at a rate of 1 to 2 Å/s under a vacuum of 10^−6^ to 10^−5^ mbar. The thickness of the film and the evaporation rate was monitored using a quartz crystal balance. The evaporation was performed with the sample holder vertically opposite the boat. This minimizes the risk of shadows on the hemispheres. However, the thickness of the film between the hemispheres was thinner than that on top due to the smaller projected area (Figure 1f). Films thicker than 50 nm were deposited in two or more evaporations. In each evaporation, the samples were rotated by 180° to avoid shadows. For example, Au films of 85 nm were evaporated in two steps: 50 nm + 35 nm. Films of 180 nm thickness were obtained in two steps: 80 nm + 100 nm. Figure 2 presents examples of PS coated spheres and hemispheres of the same diameter. The cast reproduced the topography of two-dimensional crystal very well. Distortions of the spherical shape only occurred for small spheres (400 nm).

### Optical characterization

The zeroth-order reflectance and transmittance of Au coated samples of different sizes were investigated. A WITec AlphaSNOM microscope was used for illumination and collection of the reflected light from the sample. For the illumination of samples and light collection, objectives of low numerical aperture (Nikon 10×, *NA* = 0.25) were used. The aperture angle was 15°, which restricts the detection to the zeroth-order diffraction. The illumination light source was a halogen lamp (Ocean Optics) of spectral range between 400 nm and 1000 nm. The microscope has a hot-mirror in the optical path that reflects light of wavelengths above 950 nm. The reflected light was coupled into a monochromator (Acton Research SpectraPro 300i) using a multi-mode optical fiber and detected by a liquid nitrogen cooled CCD (Princeton Instruments). Integration times of 1 s with 10 accumulations were used for each spectrum. Spectra were normalized against the reference.
